# Advances in electrochemical biosensors for the detection of tumor-derived exosomes

**DOI:** 10.3389/fchem.2025.1556595

**Published:** 2025-03-26

**Authors:** Jun Chen, Zhou Zhao, Honglin Zhu, Xiaobing Li

**Affiliations:** ^1^ School of Health Science and Engineering, University of Shanghai for Science and Technology, Shanghai, China; ^2^ Department of pathology, The Ninth People’s Hospital, Shanghai Jiao Tong University School of Medicine, Shanghai, China

**Keywords:** tumor diagnosis, tumor-derived exosomes (T-EXOs), electrochemical biosensors, liquid biopsy, biomarkers

## Abstract

Exosomes, released from diverse cells as nanoscale lipid bilayer vesicles, mediate intercellular communication and participate in various physiological and pathological processes. Thereinto, tumor-derived exosomes (T-EXOs) with molecular cargoes of parent tumor cells act as attractive biomarkers for tumor liquid biopsy. The amount of T-EXOs and their levels of contained specific proteins and nucleic acids are closely associated with cancer burden and classification. Nevertheless, the nanoscale size and relatively low abundance of exosomes, as well as complex body liquid matrix pose daunting challenges for efficient isolation and sensitive detection of T-EXOs. Biosensing as fast, convenient and accurate method, has been widely employed for the detection of biomarkers over the past decades. Among them, electrochemical sensors can sensitively detect biomarkers by measuring of the change of electrical signal caused by oxidation or reduction at the working electrode surface. This review aims to summarize the recent advance in electrochemical biosensors for quantification, and protein and RNA analysis of exosomes. Further, challenges and future perspectives for exosome-based liquid biopsy have been discussed.

## 1 Introduction

Cancer is one of the leading causes of global deaths. Early detection and precision therapy are expected to prolong survival period and improve the quality of life for cancer patients (Rebbeck et al., 2018). To date, tissue biopsy is the golden standard for cancer diagnosis, which requires tissue samples obtained by surgery and puncture for analysis (van der Laan et al., 2021). However, tissue biopsy can only provide local and temporal biological information of tumors due to obtained partial tissues and invasive sampling method, which is not conducive to early diagnosis and prognosis assessment of cancer (Lehrich et al., 2024). Alternatively, liquid biopsy can provide more comprehensive real-time information of tumor diagnosis and theray guidance by detection and analysis of circulating targets from body liquids in a non-invasive way (De Rubis et al., 2019). The common circulating targets include circulating tumor cells (CTCs), circulating exosomes and circulating tumor DNA (ctDNA) (Costa et al., 2016). Compared to CTCs and ctDNA, circulating exosomes have more advantages in liquid biopsy (Wang W. et al., 2023). First, exosomes are widely present in various body fluids, such as blood, urine, saliva, breast milk, tears and cerebrospinal fluid, and their numbers are more abundant than those of CTCs (≤100 cells/mL) (Li A. et al., 2017). Second, exosomes were more stability than ctDNA and CTCs and maintain integrity when storing in cryogenic environment (−80°C) for months (Zhou et al., 2022). Third, exosomes transport a variety of bioactive molecules that can provide a massive information about the cell of origin (Filipazzi et al., 2012). Hence, due to these unique characteristics, exosomes-based liquid biopsy shows great potential in early diagnosis of cancer and therapy guidance (Zhou et al., 2020).

As a subtype of extracellular vesicles (EVs), exosomes with a diameter of 30–180 nm are secreted from nearly all cells (Miron and Zhang, 2024). In the 1980s, exosomes were first discovered from sheep reticulocytes and named by Eberhard G. Trams and R.M. Johnstone (MacDonald et al., 2023). The formation process of exosomes is mainly divided into three steps. First, the cell membrane invades to form endosomes. The endosomal membrane further buds inward, resulting in the formation of multivesicular endosomes. Finally, the exosomes are released by the fusion of the multivesicular endosomes with plasma membrane. Exosomes were initially considered to be waste products excreted by cells (Yin et al., 2020; Han et al., 2020). Gradually, numerous evidence indicates that exosomes carry diverse cargoes from their parental cells (e.g., lipids, proteins, nucleic acids, etc.) to mediate intercellular communication and play a pivotal role in a variety of physiological and pathological processes (Xie et al., 2019). Especially, tumor-derived exosomes (T-Exos) are proved to play an important role in the genesis and development of tumors, and are considered as potential tumor biomarkers (Jiang et al., 2021). The amount of T-Exos is significant more than that of normal cells, which is conducive to tumor development (Sitkovskaya et al., 2020). In addition, T-Exos carry tumor-related specific proteins or RNAs (Kohama et al., 2019). Hence, T-Exos and tumor-related biomarkers have been largely used as circulating targets of liquid biopsy for detection of diseases and early diagnosis of cancer (Yu et al., 2023). Nevertheless, exosome-based liquid biopsy faces some daunting challenges due to small size and the low concentration of T-Exos in complex body fluids, which requires efficient isolation, intact release and sensitive detection.

Biosensing as fast, convenient and accurate method, has been widely employed for the detection of biomarkers over the past decades (Suthar et al., 2023). A biosensor is an integrated receptor-transducer device consisting of a target recognition element, a transducer, and a signal processing element, which can convert biological response into optical, electrical, mechanical, acoustic or thermal signals (Naresh and Lee, 2021). Among them, electrochemical sensors can sensitively detect biomarkers by measuring of the change of electrical signal caused by oxidation or reduction at the working electrode surface based on voltammetry, potentiometric or impedance methods, etc (Baranwal et al., 2020). More importantly, electrochemical biosensors are easy to miniaturize and integrate, which is conducive to the development of portable devices (Cao et al., 2024). Electrochemical biosensors provide a kind of sensing way with high sensitivity, fast response and simple operation for the analysis of biological samples (Vanova et al., 2021). In recent decades, various electrochemical approaches have emerged and are playing an essential role for the detection of T-EXOs (Ma et al., 2021; Bagheri Hashkavayi et al., 2020).

Over the past decade, a substantial volume of pertinent research articles has been published, highlighting the escalating interest and critical role that electrochemical biosensors play in the realm of exosome detection. These reviews summarize recent advances in exosome separation and detection methods. First, we summarize the traditional strategies for exosome isolation and determination of exosome numbers, proteins and RNA, and the important challenges they faced. Second, detection principle of exosomal biosensors based on electrochemical methods are described with each modality explained in detail. Specifically, we focus on microfluidics-based biosensors. Finally, we present new challenges and future perspectives on the development of exosome separation and detection for liquid biopsy. This review aims to provide readers with some basic knowledge of working principles of electrochemical biosensors for the exosome detection, and facilitate translation of electrochemical technologies into the clinical application of exosomes.

**FIGURE 1:**
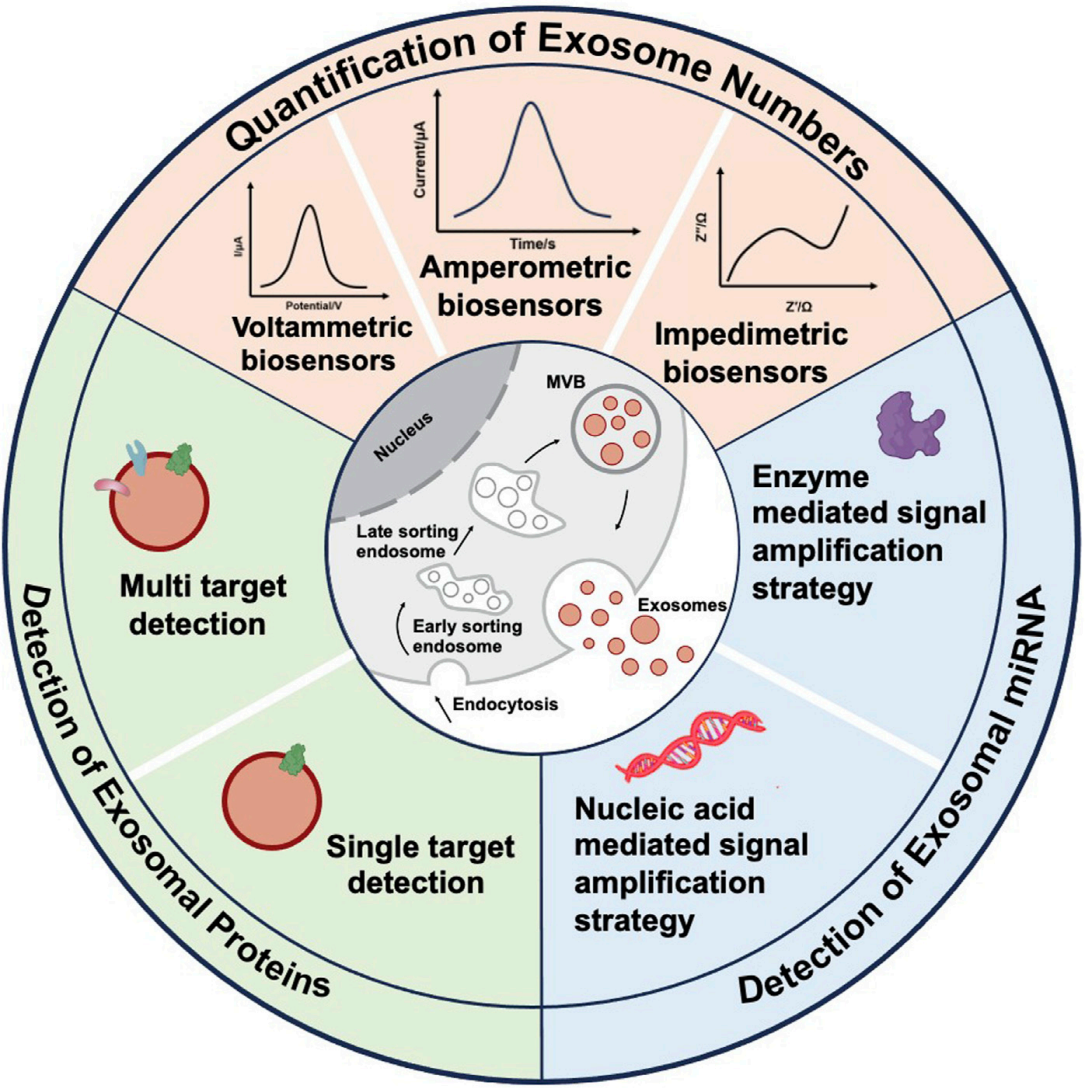
Schematic diagram of various electrochemical sensors currently used for the detection of tumor exosome numbers, tumor exosomal proteins, and tumor exosomal miRNAs.

Specifically, for the detection of tumor-derived exosomes, electrochemical biosensors are categorized based on their detection methodologies, including voltammetric, amperometric, and impedance biosensors. In the context of tumor exosomal protein detection, these biosensors are further classified into single-target and multi-target detection systems, depending on the number of analytes being measured. Regarding tumor exosomal miRNA detection, electrochemical biosensors are categorized based on signal amplification methods into nucleic acid-mediated signal amplification biosensors and enzyme-mediated signal amplification biosensors (Figure 1).

This article aims to elucidate the fundamental working principles of various electrochemical biosensors employed in exosome detection, with the ultimate goal of facilitating the translation of electrochemical technologies into clinical exosome applications. Each subsection comprehensively discusses the significant advancements and prevailing challenges associated with these sensor types.

## 2 Conventional methods for exosome detection

### 2.1 Conventional exosome quantification methods

The number of exosomes secreted by tumor cells is significant more than that of normal cells to promote the growth and metastasis of tumor cells (Fu et al., 2016). Hence, it is beneficial for the early diagnosis of cancer and assessment of tumor development to quantify the number of exosomes. Currently, the number of exosomes is mostly determined by direct particle counting methods or the standard curve of exosome concentrations *versus* output signal intensities of certain exosome surface proteins (Yahata et al., 2021; Wang et al., 2021).

Nanoparticle tracking analyzer (NTA) is a label-free optical particle quantification method. It has been widely used to determine the concentration and size distribution of exosomes by capturing the scattered light traces generated by exosomes according to the Stockes-Einstein equation (Pammi Guru et al., 2023). It has advantages of simple and rapid quantification without disrupting the structure and function of the exosomes. Nevertheless, this method is susceptible to interference from lipoproteins and protein aggregates of similar size. Adjustable resistance pulse sensing (TRPS) harnesses Coulter principle to measure the diameter and concentration of nano and submicron particles (Will et al., 2015). When exosomes suspended in electrolyte pass through nanopore chips with specific apertures, the resistance between the two electrodes inside and outside the pores rapidly changes leading to generate potential pulses. Notably, the intensity of the pulse signal is proportional to the size and concentration of exosomes. TRPS can detect the size of each exosome instead of the theoretical calculation. It requires less samples and is faster than NTA, but still interfered by lipoproteins and protein aggregates of similar size (Varenne et al., 2016).

Compared to NTA and TRPS, flow cytometry enables high - selective detection of exosome or exosome subpopulation by fluorescently labeling exosome biomarkers (Gao et al., 2021). It can quantify cells and particles as samples flow one by one through a laser beam. Fluorescence-labeled biomarkers on them are measured by scattered light and fluorescence emission signals. Conventional flow cytometers have low sensitivity and resolution for particles <500 nm, but the new A50-Micro by Apogee (United Kingdom) can classify and count vesicles with 10-nm resolution and 80-nm sensitivity (Maciejewska-Renkowska et al., 2024). It is fast, convenient and highly specific, but pricey.

ELISA with specific “sandwich” format can assess exosome amount (López-Cobo et al., 2018). Exosomes are first captured by plates with capturing antibodies, then identified by horseradish peroxidase - labeled antibodies, and finally quantified by measuring UV absorption intensity of the product. It is simple, and the concentration can be determined by the exosomal standard curve. However, commercial kits have low sensitivity with a detection limit of about 10^7^/μL.

At present, the traditional exosome detection technology is faced with the shortcomings of poor specificity or low sensitivity. To overcome the difficulties in accurately determining exosome concentration, it is urgent to develop new methods and technologies.

### 2.2 Conventional methods for exosomal protein detection

T-EXOs carry tumor biomarker proteins, and exosomal tumor-associated proteins can be biomarkers for cancer diagnosis and assessing tumor progression (Morini et al., 2023). Meanwhile, in order to further improve the accuracy of cancer diagnosis, simultaneous detection of multiple tumor-associated proteins is necessary. At present, the detection methods of exosomal proteins can be divided into labeling-based detection methods (e.g., Western blot, ELISA, Flow cytometry) and labeling-free detection methods (e.g., Mass spectrum) (Min et al., 2021).

Labeling based detection methods use fluorophore or enzyme-labeled ligands to recognize exosomal proteins and generate optical signals. Label-free methods directly lyse exosomal proteins. Western blot (WB) is an effective technique for separation and analysis of exosomal proteins with different sizes (Liu et al., 2022). Meanwhile, WB is a semi-quantitative analysis method. This method requires exosomes to be lysed, denatured, separated by SDS-PAGE, transferred, and immunoblotted. It requires a large number of samples and a long operation time (>10 h). ELISA is a faster exosomal protein quantification technology, but has the same problem as WB in detecting multiple proteins simultaneously (Zhang L. et al., 2018).

Flow cytometry based on fluorescence labeling, unlike WB and ELISA, can directly detect multiple exosomal proteins through fluorescence channels (Melnik et al., 2019). But its limited sensitivity and resolution prevent detecting particles under 200 nm. Meanwhile, this method lacks the ability to analyze individual vesicles and cannot distinguish different exosomes subgroups. Nanoflow technology based on single-molecule fluorescence detection enables quantitative characterization of single nanoparticles (Boriachek et al., 2018). However, this method requires expensive equipment and testing costs for exosomes.

The previous methods can only detect the labeled protein. Mass spectrometry allows high-throughput protein determination, but exosomes need enzymatic digestion and peptide separation (Maybruck et al., 2017). However, due to the influence of high-abundance proteins and the lack of specific markers, low abundance of protein is easily masked, leading to challenges in detection. At the same time, this method requires expensive equipment and takes a long time.

In a word, traditional protein assays of exosomes are not suitable for the clinical application. The high throughput analysis and multi-target detection of exosomal proteins still face great challenges. Meanwhile, it is necessary to develop new technologies with low sample volumes and simple processing for biomedical applications.

### 2.3 Conventional methods for exosomal microRNAs detection

In addition to protein cargoes, exosomes carry different types of nucleic acids, including DNA, mRNAs, microRNAs (miRNAs) and non-coding RNAs (Ye et al., 2022). Among them, miRNAs are a type of endogenous non-coding small RNAs of ∼17–24 nucleotides (nt), mediating post-transcriptional regulation (Zheng et al., 2021). Notably, some miRNAs can be selectively and preferentially loaded into T-EXOs in response to their cell of origin. MiRNAs from T-EXOs regulate biological processes, involve in tumor growth and metastasis, and are tumor diagnostic markers (Zheng et al., 2021). Accurate detection of exosomal miRNAs is significant for disease diagnosis and monitoring. However, their small size, low abundance and high sequence homology among family members bring daunting challenges for detection.

So far, several detection methods for exosomal miRNAs have been constructed, mainly including Northern blot, reverse transcription-polymerase chain reaction (RT-PCR) and miRNA microarrays. Northern blot, the gold standard, involves agarose gel electrophoresis of denatured miRNAs, transfer to a membrane, and detection with a labeled RNA probe (Wery et al., 2024). It can detect mature miRNAs and their precursors, but has low throughput, low sensitivity, and is time-consuming. RT-PCR converts RNA to cDNA and amplifies it (Chen et al., 2005). This method has good sensitivity and specificity, but RT-PCR is complicated, costly, and has unstable results and potential false positives. MiRNA microarray is a high-throughput technology for gene expression abundance detection (Roy et al., 2016). However, this method cannot detect miRNAs that are too short and miRNAs with a low copy number and are specific for detecting miRNAs with similar sequences.

Overall, these conventional detection methods have more or less certain limitations. Therefore, many researchers are dedicated to finding a cheap, fast, and highly accurate and sensitively method for miRNA detection.

## 3 Electrochemical biosensors for quantification of exosome numbers

To overcome drawbacks of conventional exosome quantification assay, biosensors have been developed to convert exosome number information into measurable physicochemical signals. Among them, electrochemical biosensors include signal transduction elements and biometric elements, which can convert the biological information detected by the biosensing elements into electrical signals (Wang et al., 2022). Electrochemical biosensors quantify exosome concentration by detecting changes in electrical signals. These changes result from alterations in the electrochemical reaction on the electrode surface due to interactions between recognition molecules and exosomes (Su et al., 2022). Electrochemical biosensors are classified based on detection methods into voltammetric, amperometric, and impedimetric biosensors. Electrochemical biosensors with merits of high sensitivity, low cost and good portability have been widely employed for the analysis of exosome quantities. This part will summarize recent advances in electrochemical biosensors for the quantification of exosome numbers (Table 1).

**TABLE 1:** Comparison of the electrochemical biosensors for quantitative determination of exosomes.

Biosensing strategy	Isolation approach	LOD (particles/μL)	Linear range (particles/μL)	References
Voltammetric	Ultracentrifugation	70	10^3^–1.2 × 10^5^	Dong et al. (2018)
Voltammetric	Ultracentrifugation	100	10^2^–10^7^	Boriachek et al., 2017
Voltammetric	Ultracentrifugation	Ā	N/A	Zhou Y. G. et al., 2016
Voltammetric	Ultracentrifugation	20.9	10^2^–10^9^	Wang et al., 2017
Voltammetric	Ultracentrifugation	6/mL	0.1–10^4^	Jiang et al., 2024
Voltammetric	Microfluidic	1.0 × 10^3^	10^3^–10^6^	Zhou et al. (2016b)
Voltammetric	Microfluidic	4.39	7.61 × 10^4^–7.61 × 10^8^	Xu et al. (2018)
Voltammetric	Microfluidic	17	10^2^–10^9^	Kashefi-Kheyrabadi et al. (2020)
Amperometric	Ultracentrifugation	200	2.0 × 10^2^–10^6^	Ximena et al. (2016)
Amperometric	Ultracentrifugation	96	1.12 × 10^3^–1.12 × 10^8^	An et al. (2019)
Amperometric	Ultracentrifugation	0.5	2.4–10^4^	Zhang et al. (2021a)
Impedimetric	Ultracentrifugatio	190	N/A	Li et al. (2017b)
Impedimetric	Ultracentrifugatio	20/mL	N/A	Irani et al. (2023)
Impedimetric	Ultracentrifugatio	77/mL	0.1–10^6^	Kilic et al. (2018)
Impedimetric	Ultracentrifugatio	0.87	10^2^–10^9^	Sazaklioglu et al. (2024)
Impedimetric	Microfluidic	42	50–10^5^	Wang et al. (2023b)

### 3.1 Voltammetric biosensors

**FIGURE 2:**
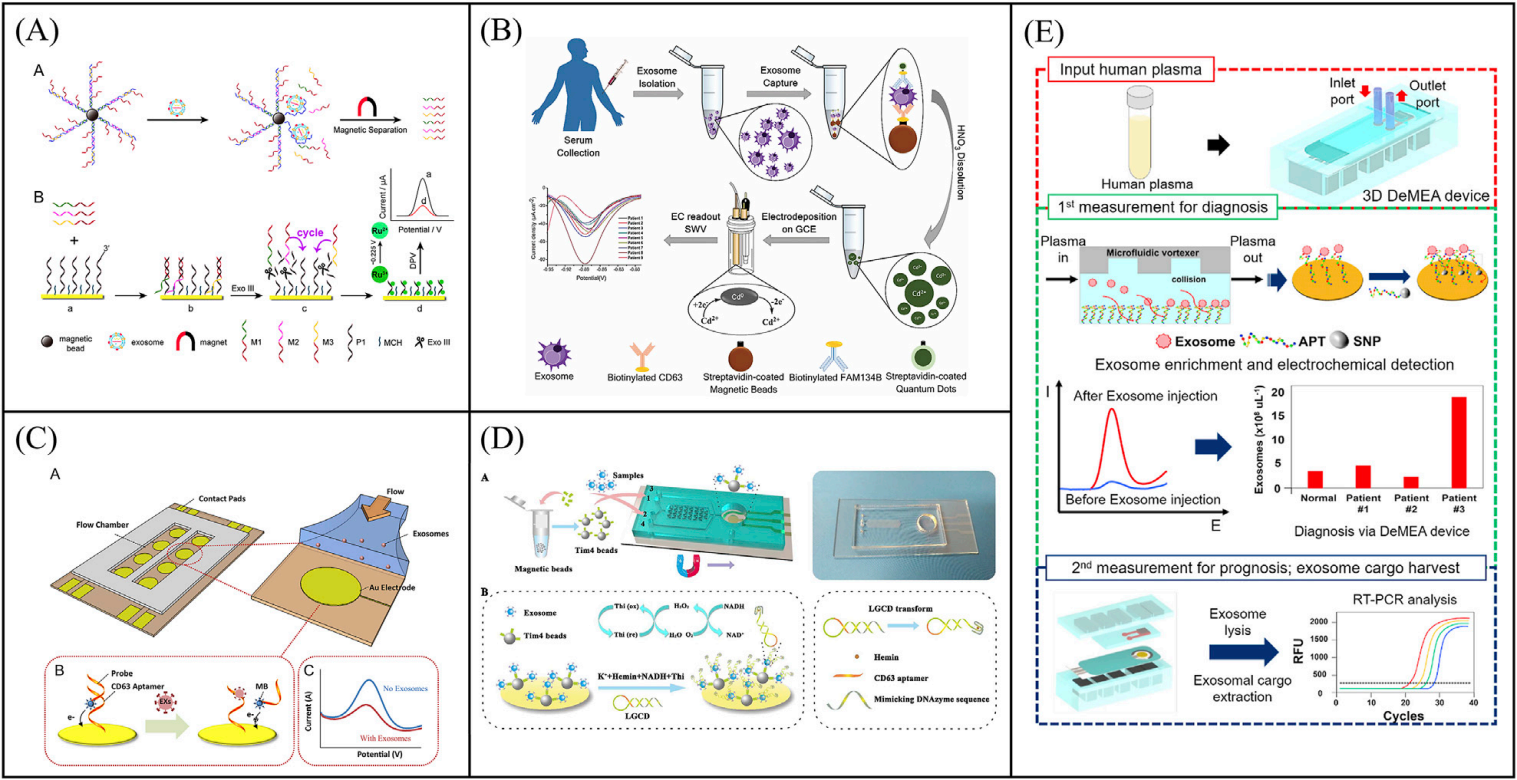
Examples of voltammetric biosensors for exosome quantity detection. **(A)** An electrochemical sensor for sensitive detection of exosomes based on multiple DNA release and enzyme cycle amplification. Reprinted with permission from Ref. Dong et al. (2018); Copyright (2018) with permission from American Chemical Society. **(B)** Working principle of a sandwich structure-based electrochemical sensor for the detection of T-EXOs. Reprinted with permission from Ref. Boriachek et al., 2017; Copyright (2017) with permission from Royal Society of Chemistry. **(C)** Working principle of a microfluidic chip electrochemical sensor based on aptamer recognition for exosome detection. Reprinted with permission from Ref. Zhou et al. (2016b); Copyright (2016) with permission from Elsevier. **(D)** A two-stage microfluidic platform (ExoPCD chip) for the direct capture of exosomes and *in situ* electrochemical analysis can effectively circumvent the complex manufacturing processes and high costs associated with gold electrode-integrated microfluidic chips. Reprinted with permission from Ref. Xu et al. (2018); Copyright (2018) with permission from American Chemical Society. **(E)** A detachable microfluidic device with electrochemical adapter sensor (DeMEA) for ultra-sensitive detection of tumor exosomes. Reprinted with permission from Ref. Kashefi-Kheyrabadi et al. (2020); Copyright (2020) with permission from Elsevier.

Voltammetric analysis relies on the measurement of current as a function of electrode potential. It has been widely used in electrochemical biosensors due to high sensitivity and simple operation. In voltametric biosensors, exosomes are recognized by recognition ligands and quantified by measuring the current intensity of electrochemical probe molecules at the electrode. For example, Dong et al. designed a voltametric-based electrochemical sensor for sensitive detection of exosomes (Figure 2A). First, three kinds of messenger DNA (mDNAs) were hybridized with the PSMA aptamer−magnetic bead. When exosomes are present, mDNAs were released due to exosomes bind to PSMA aptamer and were further captured by the probe DNAs immobilized on a gold electrode (Dong et al., 2018). Subsequently, exonuclease III was employed to assist in target recycling for signal amplification. The exosomes were quantified through the determination of the peak current of Ru(NH_3_)_6_^3+^ adsorbed on the DNA using differential pulse voltammetry (DPV) method. This method, based on multiple DNA release and enzyme cycle amplification, enabled a limit of detection (LOD) of 70 particles/μL. Compared with single-ligand recognition, dual-ligand recognition of exosomes using a sandwich structure can significantly improve analytical specificity. Moreover, coupled with signal amplification strategies, sandwich structure-based immunoassay methods can achieve sensitively quantitative analysis of exosomes. For example, Boriachek et al. constructed a sandwich structure-based electrochemical sensor for the detection of T-EXOs (Figure 2B) (Boriachek et al., 2017). Exosomes were first captured by magnetic beads modified with antibodies against tetranemmembrane proteins, and then specific antibodies (FAM134B for colon and HER2 for breast cancer) labeled with CdSe quantum dots recognized proteins on the surface of exosomes. Finally, CdSe quantum dots as signal amplifier were dissolved using acid, and Cd^2+^ was detected by square-wave anodic stripping voltammetry (SWASV). The linear range of exosome detection by this sensor was 1 × 10^2^ to 1 × 10^7^ particles/μL, and the LOD was 100 particles/μL. Meanwhile, a biosensor based on the sandwich strategy also allows for the direct capture of exosomes on electrodes for detection, simplifying experimental operations and reducing the sample volume required. For example, Zhou et al. presented an electrochemical sensor based on a sandwich structure for the direct capture and detection of PCa exosomes on electrodes (Zhou Y. G. et al., 2016). Exosomes captured by aptamer-functionalized gold electrodes were further recognized by silver nanoparticles (AgNPs) modified with EpCAM aptamer and copper nanoparticles (CuNPs) modified with PSMA aptamer. The signals from AgNPs and CuNPs were directly read out due to their oxidation potentials using the linear sweep voltammetry (LSV) technique. This biosensor achieved an LOD of 50 particles/sensor. However, single-stranded DNA immobilized on the electrode can easily become entangled with each other, reducing the sensitivity of exosome detection. To overcome this challenge, Wang’s group developed a nano-tetrahedron-assisted aptamer sensor for the direct capture and detection of exosomes (Wang et al., 2017). Since nano-tetrahedron could maintain the spatial orientation of aptamers and improve biomolecular recognition, this sensor has a 100-fold higher detection sensitivity and a wide detection linear range (1 × 10^2^ to 1 × 10^9^ particles/μL) with an LOD of 20.9 particles/μL. In addition, Jiang et al. developed an aptamer-carrying tetrahedral DNA (Apt-TDNA) microelectrode sensor with a poly-dopamine (PDA) coating, aimed at ultra-sensitive electrochemical detection of T-EXOs (Jiang et al., 2024). The stable rigid structure and orientation of Apt TDNA ensured effective capture of exosomes, while the PDA coating effectively amplified electrical signals. The sensor achieved sensitivity at the single particle level and was tested using the DPV method with an LOD of 6 × 10^3^ particles/μL. Voltammetric-based electrochemical biosensors show great potential for quantitative exosomes due to high sensitivity.

With the development of manufacturing processes, electrodes can be integrated into microfluidic chips to achieve detection of exosomes. Microfluidics-assisted electrochemical biosensors address the limitations of conventional sensors, such as long detection times and low efficiency due to the large volume of detection solution. At the same time, microfluidics-assisted electrochemical biosensors are more suitable for clinical applications due to its portability. For instance, Zhou et al. developed a microfluidic chip electrochemical sensor that used aptamer recognition for exosome detection (Figure 2C) (Zhou Q. et al., 2016). The gold electrode surface of the microfluidic chip was functionalized with CD63 aptamers, and a DNA detection chain labeled with methylene blue was hybridized with the CD63 aptamers. Upon exosome capture, which triggered the release of the beacon molecule, the redox current detected by the electrode decreases. Further, exosomes were determined by the change of current with an LOD of 1 × 10^3^ particles/μL. To circumvent the complex manufacturing process and high costs associated with gold electrode-integrated microfluidic chips, Xu et al. developed a two-stage microfluidic platform (ExoPCD chip) that integrated serum exosome separation using Y-shaped microcolumns with *in situ* electrochemical analysis using an indium tin oxide (ITO) slice, facilitating rapid exosome separation and downstream analysis of complex biological fluids (Figure 2D) (Xu et al., 2018). Tim4-modified magnetic beads were used to capture exosomes expressing phosphatidylserine. CD63-positive exosomes can open the original single-stranded DNA hairpin and form G-tetraposomes with the help of heme chloride as NADH oxidase for signal transduction. This microfluidics-assisted electrochemical biosensor, which did not require exosome-labeled electrochemical probes, achieved an LOD of 4.39 particles/μL for exosomes. However, most microfluidics-assisted electrochemical biosensors utilize planar electrodes, leading to low exosome capture efficiency. To overcome this problem, Kashefi-Kheyrabadi et al. developed a detachable microfluidic device with electrochemical adapter sensor (DeMEA) for ultra-sensitive detection of tumor exosomes (Figure 2E) (Kashefi-Kheyrabadi et al., 2020). A nanocomposite was first applied on the electrode surface to improve the immunocapture efficiency of exosomes. Microfluidic vortices were then integrated with electrochemical sensors *via* detachable clips to increase collisions between exosomes and sensing surfaces. The electrode modified with EpCAM aptamer was used to capture and detect tumor exosomes by DPV method with a LOD of 17 particles/μL. Subsequent harvest of exosomes were used for downstream analysis by real-time polymerase chain reaction. DeMEA offers a promising approach for sensitive exosome detection using microfluidic-assisted biosensors. In conclusion, a low-cost, portable and highly sensitive electrochemical chip integrated microfluidic platform has generated a lot of interest as an excellent tool for analyzing exosome quantity.

### 3.2 Amperometric biosensors

**FIGURE 3:**
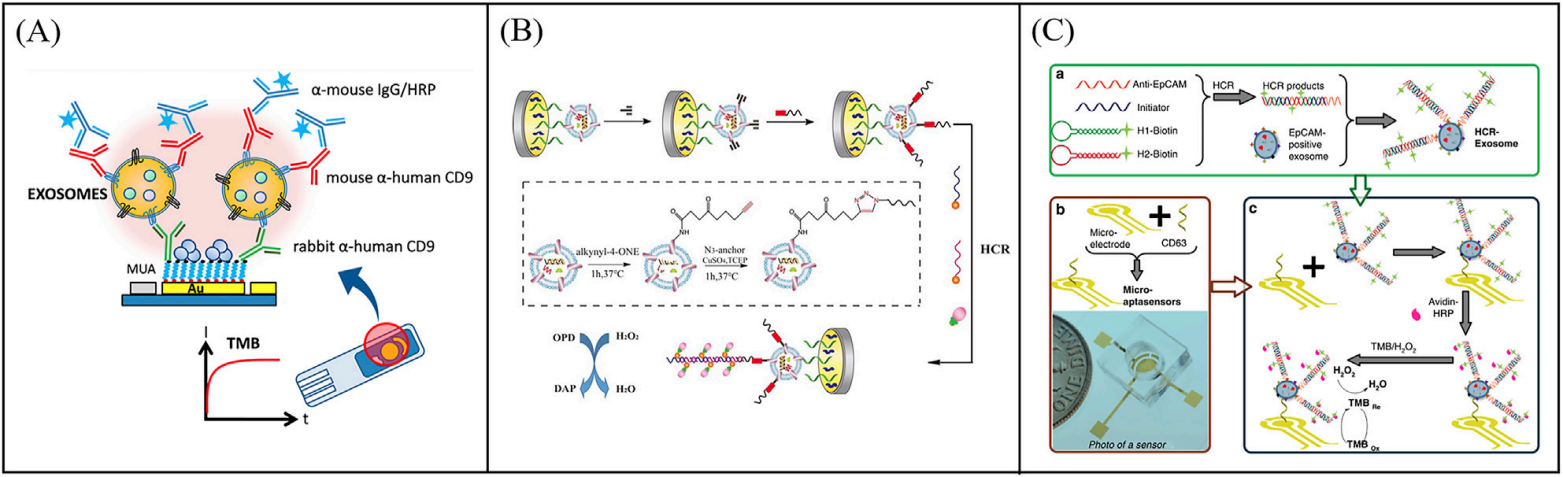
Examples of amperometric biosensors for exosome quantity detection. **(A)** Schematic illustration of a sandwich immunoelectrochemical sensor based on HRP-labeled antibody for detection of exosomes. Reprinted with permission from Ref. Ximena et al. (2016); Copyright (2016) with permission from American Chemical Society. **(B)** Schematic illustration of the electrochemical aptasensor for exosome detection based on click chemistry and HCR for signal amplification. Reprinted with permission from Ref. An et al. (2019); Copyright (2019) with permission from Elsevier. **(C)** Working principle of a micropatterned electrochemical for the detection of exosomes based on the micropatterned electrodes and HCR dual-amplification strategy, aiming to meet the clinical demand for portable biosensors. Reprinted with permission from Ref. Zhang et al. (2021a); Copyright (2021) with permission from Springer Nature.

Amperometric analysis methods rely on the measurement of electrode current as a function of time. Enzymes, such as HRP, are common employed to catalyze redox reaction in amperometric electrochemical biosensors. For enzyme-based amperometric biosensors, the efficiency and sensitivity of sensors depend on the electrode modification, type of enzyme, and substrates. In 2016, Doldan’s group developed a sandwich immunoelectrochemical sensor based on HRP-labeled antibody, affording an LOD of 200 exosomes/μL (Figure 3A) (Ximena et al., 2016). To improve the enzyme load and increase the detection sensitivity, An et al. constructed an electrochemical aptasensor *via* DNA hybridization chain reaction for signal amplification detection of exosomes (Figure 3B) (An et al., 2019). CD63 aptamer was first immobilized on electrodes for capturing exosomes. 4-oxo-2-nonenal alkyne (alkynyl-4-ONE) molecules, functionalized lipid electrophiles, were employed for the nonspecific conjugation of protein on the surface of exosomes *via* the reaction of amino and aldehyde groups. Azide-labeled DNA as an anchor was conjugated to the exosomes by copper (I)-catalyzed click chemistry. Subsequently, the DNA hybrid chain formed by self-assembly can be loaded with more HRP for signal amplification. The concentration of exosomes can be quantified by measuring the reduction current of the product 2,3-diaminophenazine of phenylene diamine. The method shows a low LOD of 96 particles/μL and a wide linear range of 1.12 × 10^2^–1.12 × 10^8^ particles/μL. To develop portable biosensors to meet clinical detection needs, Zhang et al. reported a micropatterned electrochemical aptasensor for the detection of exosomes based on the micropatterned electrodes and HCR dual-amplification strategy (Figure 3C) (Zhang W. et al., 2021). The exosomes were captured by CD63 aptamer-functionalized electrodes and recognized by EpCAM aptamers-assisted HCR products containing lots of HRP. Subsequently, TMB reduction current was measured for exosome quantification. The sensors achieved a wide detection linear range from 2.4 to 1 × 10^4^ particles/μL with an LOD of 0.5 particles/μL. However, enzyme-based amperometric biosensors suffer from the reduction of enzyme activity from the environment (e.g., pH, temperature, ionic strength, *etc.*). It is highly desirable to improving the stability of enzymes or develop new catalytic materials for accelerating the development of amperometric biosensors.

### 3.3 Impedimetric biosensors

**FIGURE 4:**
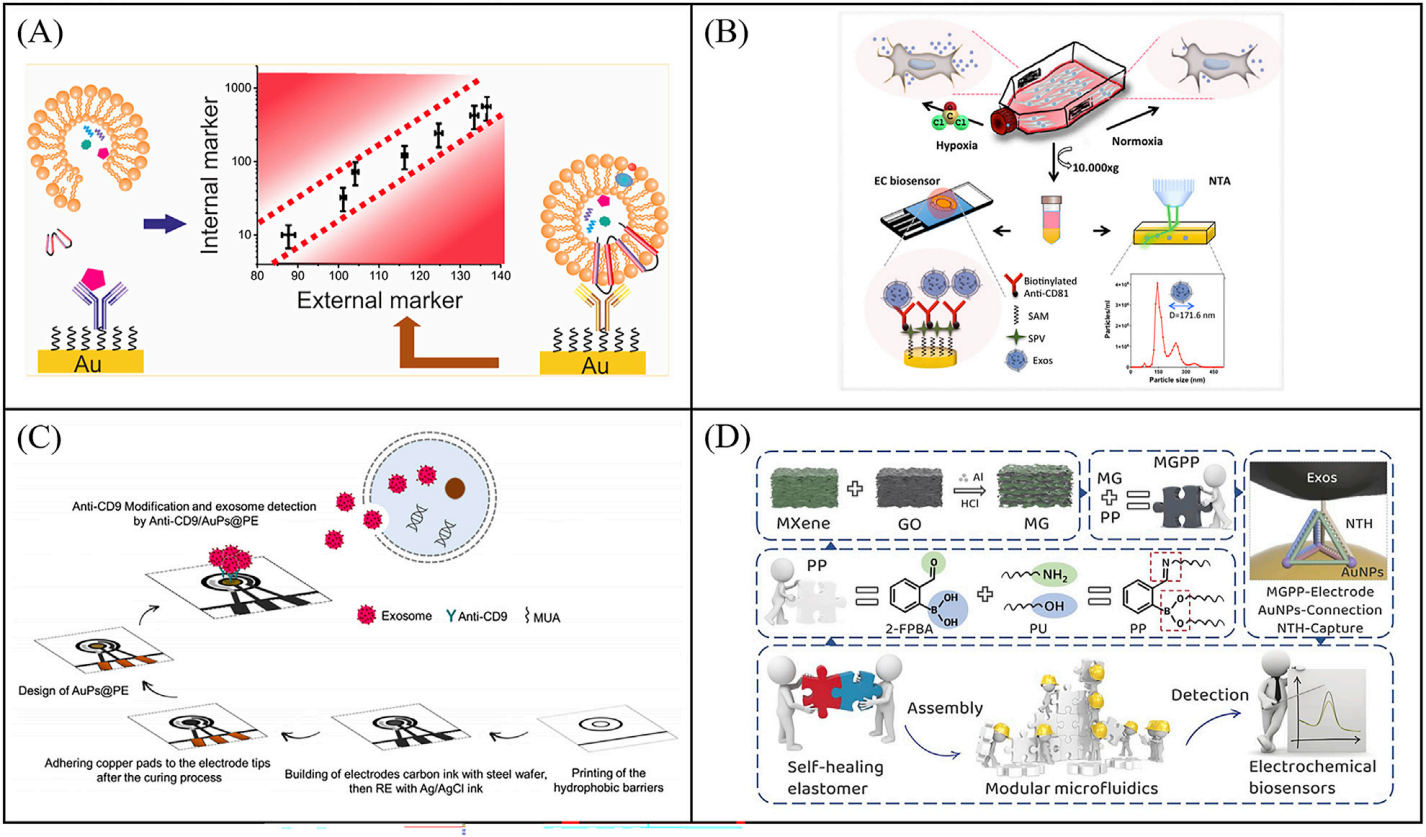
Impedimetric biosensors for exosome quantity detection. **(A)** An impedimetric biosensors to quantify exosomes, the exosomes were captured by CD81 antibody-functionalized Au electrodes and quantified by detecting interfacial impedance changes. Reprinted with permission from Ref. Irani et al. (2023); Copyright (2022) with permission from Elsevier. **(B)** Schematic illustration of an impedimetric biosensors for label-free detection of MCF-7 cells derived exosomes of hypoxic and normoxic conditions Kilic et al. (2018). **(C)** Working principle of a paper-based immunosensor based on electrochemical impedance spectroscopy for exosome detection, aimed at developing more portable sensors. Reprinted with permission from Ref. Sazaklioglu et al. (2024); Copyright (2024) with permission from Springer Vienna. **(D)** The construction method of the self-healing conductive elastomer MG/PP and the strategy for utilizing MG/PP in the development of microfluidic systems Wang et al. (2023b).

Impedimetric biosensors detect analytes by measuring the impedance value of the electrode surface under a small amplitude of *alternating current* (AC) voltage of different frequencies. When the exosomes are captured on the electrode, it prevents the transfer of the electroactive probe (K_3_ [Fe(CN)_6_] and K_4_ [Fe(CN)_6_]) to the electrode surface thus causing a change in impedance. Exosome concentrations can be measured by impedance changes. Impedimetric-based biosensors combines the advantages of impedimetric without labeling and immunoassay of high selectivity for the analysis of exosomes. Li et al. reported an impedimetric biosensors to quantify exosomes (Li Q. et al., 2017). The exosomes were captured by CD81 antibody-functionalized Au electrodes and quantified by detecting interfacial impedance changes with an LOD of 190 particles/μL (Figure 4A). The internal exosome-specific markers can also be further analyzed after cleavage. To further enhance the capture efficiency of exosomes on electrode, Irani et al. developed a highly sensitive electrochemical biosensor featuring a gold nanoisland (Au-NIs) structure (Irani et al., 2023). They modified the FTO electrode with gold nanostructures through physical vapor deposition, thermal annealing and electrochemical deposition. The gold nanostructures, characterized by their high surface-to-volume ratio, which allows more CD-151 antibodies to be fixed to capture exosomes. The biosensor was tested using the EIS method, and the detection limit was ultimately proved to be 2 × 10^4^ particles/μL. To develop more portable sensors, killic et al. reported an impedimetric biosensors using screen printed electrode for label-free detection of MCF-7 cells derived exosomes of hypoxic and normoxic conditions (Figure 4B) (Kilic et al., 2018). The exosomes were first captured by CD81 antibody-functionalized Au electrodes and further measured *via* differential pulse voltammetry (DPV) and electrochemical impedance spectroscopy (EIS). EIS-based assays with an LOD of 7.7 × 10^4^particles/μL showed higher sensitivity than DPV with a LOD of 3.79 × 10^5^ particles/μL in the working linear range of 10^5^–1.0 × 10^12^ particles/μL. Furthermore, Sazaklioglu et al. devised a paper-based immunosensor to identify exosomes by electrochemical impedance spectroscopy (Figure 4C) (Sazaklioglu et al., 2024). The sensor comprised gold particles modified on a paper electrode, which was then coupled with a CD9 antibody (Anti-CD9/AuPs@PE) for exosome detection. The sensor provided a convenient, low-cost, biodegradable alternative with a detection limit of 8.7 × 10^5^ particles/μL. The integrated structure of screen-printed electrodes makes it difficult to add new functions, while the modular microfluidic assembly and preparation systems can effectively overcome the above problems. Wang et al. constructed a self-healing conductive elastomer MG/PP by combining a metal *in-situ* reduced MG conductive network with a hard room temperature self-healing elastomer substrate PP (Figure 4D) (Wang M. et al., 2023). And proposed a strategy to use the self-healing properties of MG/PP for modular microfluidics: MG/PP served as a self-supporting electrode and utilized tetrahedral DNA nanostructures (TDN) as recognition molecules for high-sensitivity detection of colorectal cancer exosomes. The biosensor was tested exosomes with a wide linear detection range (50 to 10^5^ particles/μL) and an LOD of 42 particles/μL. More importantly, this method provides a simple approach for constructing microfluidic systems by combining modules to precisely position microfluidic components with just a few simple operations, without the need for external interfaces. Therefore, microfluidic engineering can be carried out efficiently by anyone. In the future, the scalable modular microfluidic devices can be further strengthened to make the modular component library larger and more suitable for the design of complex microfluidic system design. All in all,impedimetric-based immunobiosensors techniques without labeling show great potential in the quantitative determination of exosomes.

Electrochemical biosensors have emerged as powerful analytical tools for exosome detection, utilizing diverse electrochemical detection methods. By integrating signal amplification strategies and the molecular recognition capabilities of aptamers or antibodies, these electrochemical biosensors demonstrate exceptional sensitivity and specificity in detecting tumor exosomes. The integration of microfluidic technology has further enabled the development of portable electrochemical platforms, significantly enhancing their potential for clinical translation. However, challenges remain in terms of detection throughput, cost-effectiveness, and interference resistance in complex biological matrices. It is necessary to further develop more stable probe immobilization methods, high-throughput portable devices, and low-cost detection solutions, while enhancing anti-interference capability in complex samples, to promote their widespread application in clinical diagnosis and point-of-care testing.

## 4 Electrochemical biosensors for the detection of exosomal proteins

Tumor-derived exosomes carry tumor-specific marker. It is worth noting that a type of cancer often has multiple tumor markers, and simultaneous detection of multiple protein targets can effectively improve the accuracy of cancer diagnosis. Meanwhile, cancer is a heterogeneous disease, and there are various subtypes with different clinical behaviors (e.g., invasive ability, drug resistance, *etc.*) (Bianchini et al., 2016). The analysis of expression level of exosome biomarkers is effective for clinical diagnostic and classification due to the different expression in different subtypes (Li et al., 2023). However, the conventional assays mentioned above for exosomal proteins have many drawbacks, such as large volumes of samples and laborious procedures, which cannot meet the clinical needs. Electrochemical biosensors have shown great potential in multi-target protein analysis of exosomes due to the development of multi-pathway electrodes. At present, microfluidics-assisted electrochemical sensor is rarely used to detect exosomal proteins, which may be due to the complex processing and high cost of electrochemical microfluidic chips, and it is difficult to realize the simultaneous detection of multiple proteins. Table 2 summarizes the recent advances in electrochemical biosensors for the detection of exosomal proteins with the focus on the detection method, exosome isolation approach, type of disease, detection target and detection limit.

**TABLE 2:** Comparison of the electrochemical biosensors for exosomal proteins detection.

Target number	Exosome isolation approach	Disease	Target protein	LOD (particles/μL)	References
Single target	Ultracentrifugation	Breast cancer	HER2	4.7 × 10^5^	Yadav et al. (2017)
Single target	Ultracentrifugation	H460	CD63	N/A	Wei et al. (2013)
Single target	Selectively captured	Hela Cells	CD6, EpCAM	N/A	Huang et al. (2023)
Multi target	Ultracentrifugation	Prostate cancer	EpCAM, PSMA	50/sensor	Zhou et al. (2016a)
Multi target	Ultracentrifugation	Breast cancer	EpCAM, HER2	3.4 × 10^3^ exosomes/μL	Zhang et al. (2023)
Multi target	Ultracentrifugation	Breast cancer	EpCAM, MUC1, HER2, CEA	N/A	An et al. (2020)
Multi target	Ultracentrifugation	various tumor cells	EGFR, CEA,EpCAM	N/A	Wang et al. (2023c)
Multi target	Magnetic separation	Ovarian cancer	EpCAM, CD24, CA125, HER2, MUC18, EGFR	3 × 10^4^	Jeong et al. (2016)
Multi target	Microfluidic	Breast cancer	PMSA, EGFR, CD81, CEA	1 × 10^4^	Wang et al. (2023d)

With the development of manufacturing technology, multi-channel electrodes with different materials and different shapes have greatly enriched the application of electrochemical biosensors. At present, electrochemical sensor is a powerful tool for multi-target analysis of biomarkers.

### 4.1 Single target detection of exosomal proteins

**FIGURE 5:**
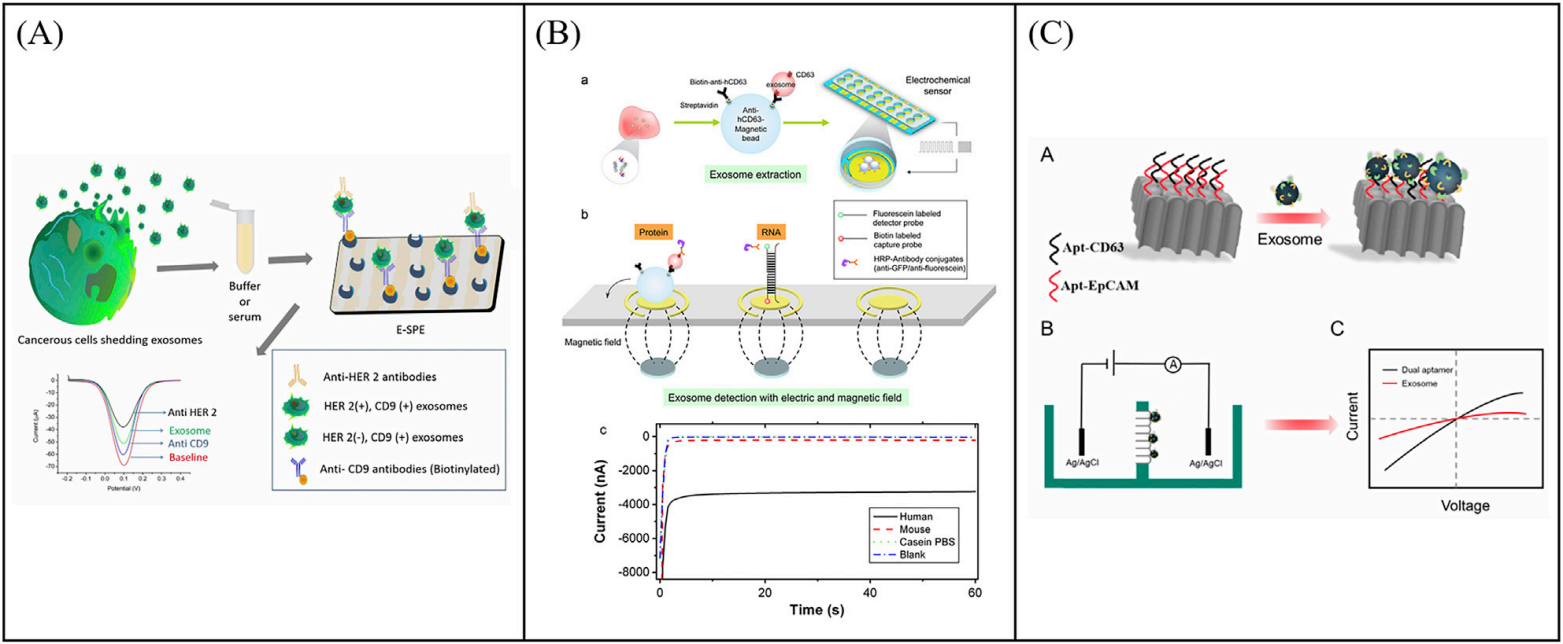
Examples of electrochemical sensors for single target detection of exosomal proteins. **(A)** Schematic illustration of an electrochemical sensor using extra avidin-modified screen-printed electrodes (E-SPE) for directly quantify the disease exosomes. Reprinted with permission from Ref. Yadav et al. (2017); Copyright (2017) with permission from John Wiley and Sons Ltd. **(B)** Working principle of an electrical field-induced release and measurement (EFIRM) biosensor for exosomal protein detection. Reprinted with permission from Ref. Wei et al. (2013); Copyright (2013) with permission from Elsevier. **(C)** An electrochemical sensor based on bivalent aptamer functionalized nanochannels for detection exosomal proteins Huang et al. (2023).

Traditional electrochemical sensors initially focus on the detection of single target proteins of exosomes. In 2016, Yadav et al. report a DPV measurement method to directly quantify the disease exosomes using extra avidin-modified screen-printed electrodes (E-SPE) (Yadav et al., 2017). The exosomes were captured by E-SPE modified CD9 antibodies and further recognized by human epidermal growth factor receptor 2 (HER2) antibodies (Figure 5A). The addition of exosomes on E-SPE prevented effective transfer of [Fe(CN)_6_]^4-/3-^ across the electrode, resulting in a decrease in DPV current response. This method showed excellent specificity for HER2 (+) exosomes from breast cancer cells. To further expand the detection throughput of exosomal proteins, Wei et al. developed a pioneer work using an electrical field-induced release and measurement (EFIRM) biosensor for exosomal protein detection based on an array chip with 16 bare gold electrodes (Figure 5B) (Wei et al., 2013). In this work, exosomes were directly isolated from serum or saliva *via* the CD63 antibody-modified magnetic beads and collected on the electrodes by a magnet. Then the EFIRM biosensor was detected the signal generated by reaction between the HRP-labeled target exosomal proteins and TMB. the EFIRM biosensor opens up a novel facet of molecular diagnostics in that the non-invasive detection of tumor-specific constituents of protein, microRNA, mRNA as well as tumor-specific mutation profiles maybe feasible in saliva. To further improve the capture stability and detection specificity of exosomes, Huang et al. proposed an electrochemical sensor based on bivalent aptamer functionalized nanochannels (Huang et al., 2023). They immobilized aptamers targeting CD63 and EpCAM simultaneously on the nanochannels to construct bivalent aptamer functionalized nanochannels (Figure 5C). When the target exosomes pass through, they can be recognized and selectively captured in a bivalent synergistic manner by functionalized nanochannels. This method provides a highly specific, sensitive, and accurate method for the detecting of T-EXOs.

### 4.2 Multi target detection of exosomal proteins

Relying on the analysis of single exosomal protein cannot accurately identify the cancer. In order to further realize the simultaneous detection of multiple proteins, Zhou et al. presented an electrochemical sensor using a microfabricated chip with multi-gold electrodes for the detection of PCa exosomes (Zhou Y. G. et al., 2016). Electro-oxidation of the labeled MNPs can recognize EpCAM and PSMA proteins expressed by exosomes. In addition, Zhang et al. constructed a one-step multi analytical electrochemical aptamer sensor based on a multi probe recognition strategy for the analysis of breast cancer exosomes (Zhang et al., 2023). Methylene blue (MB) functionalized HER2 aptamers and ferrocene (Fc) functionalized EpCAM aptamers were used as signal units to modify respectively on gold nanoparticles (AuNPs). By recognizing three aptamers through exosomes, two AuNPs modified with MB and Fc could be specifically captured on the electrode. Then, a one-step multiplex analysis of extracellular vesicles was achieved by detecting two independent electrochemical signals of nanoparticles. This method showed potential in identifying HER2 positive breast cancer exosomes.

**FIGURE 6:**
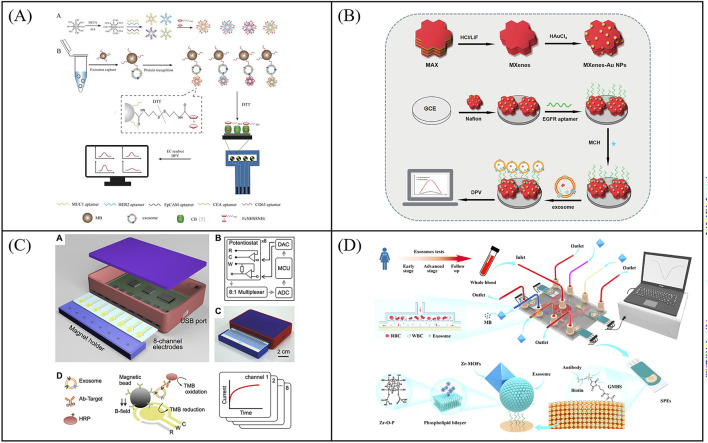
Examples of electrochemical sensors for multi target detection of exosomal proteins. **(A)** Schematic illustration of a magneto-mediated electrochemical biosensor based on host–guest recognition for simultaneous analysis of exosomal proteins. Reprinted with permission from Ref. An et al. (2020); Copyright (2020) with permission from American Chemical Society. **(B)** An electrochemical biosensor based on MXenes AuNPs modified to evaluate differential expression of EGFR, CEA, and EpCAM proteins in T-EXOs. Reprinted with permission from Ref. Wang et al. (2023c); Copyright (2023) with permission from Elsevier. **(C)** An integrated magnetic electrochemical exosome (iMEX) biosensor for multiplexed exosomal protein detection Jeong et al. (2016). **(D)** Working principle of the filter-electrochemical microfluidic chip (FEMC) for analysis of exosomal proteins. Reprinted with permission from Ref. Wang et al. (2023d); Copyright (2023) with permission from Elsevier.

To further improve the throughput of protein detection, multi-target and highly sensitive detection platforms have gradually sprung up. For example, An et al. designed a magneto-mediated electrochemical biosensor based on host-guest recognition for simultaneous analysis of breast cancer exosomal proteins using screen-printed carbon electrode (Figure 6A) (An et al., 2020). The electroactive tags (with the ability of specific exosomal proteins identification and signal output) were first formed a sandwich structure with exosomes captured by CD63 aptamer-modified magnetic beads. Then the electroactive molecules released from the electroactive tags after dithiothreitol treatment, and formed the stable complexes with the graphene oxide-cucurbit (Li A. et al., 2017) modified screen-printed carbon electrode. Exosomal proteins were further quantified by the oxidation current signal of electroactive molecules. The electrochemical biosensor was able to sensitively and simultaneously detect four kinds of exosomal proteins from different breast cancers. To further improve the capture efficiency of extracellular vesicles on electrodes, Wang et al. developed an electrochemical biosensor based on MXenes AuNPs modified to evaluate differential expression of EGFR, CEA, and EpCAM proteins in T-EXOs (Figure 6B) (Wang Z. et al., 2023). Gold nanoparticles (AuNPs) combined with MXenes to form MXenes AuNPs composite nanomaterials, which have a high surface volume ratio, unique conductivity, and adsorption capacity, and could immobilize more ligands to capture T-EXOs. This sensor could sensitively detect differential expression of tumor biomarkers in T-EXOs (including A549, MCF-7, PC-3, and HeLa), which was helpful for early and accurate diagnosis of tumor. To develop more portable sensing devices, Jeong et al. reported a portable integrated magnetic electrochemical exosome (iMEX) biosensor for multiplexed exosomal protein detection (Figure 6C) (Jeong et al., 2016). The iMEX biosensor used multichannel design and offered simultaneous detection of eight markers within 1 h and each marker only required 10 μL plasma. It is anticipated that multi-target detection can be conducive to improve the accuracy of diagnosis, avoid missed and misdiagnosed, and be beneficial to avoid other more expensive examinations, thereby reducing the waste of medical resources and reducing medical costs. However, the recognition and isolation method based on antibody magnetism ignores the small difference of common surface proteins of exosomes, which may lead to the loss of some exosomes. To solve this problem, Wang et al. developed a new filter electrochemical microfluidic chip (FEMC), which can directly detect and classify breast cancer (BC) in the whole blood. In this method, exosomes were enriched through a dual filtration system and then guided through a curved channel onto four screen printed electrodes (Figure 6D) (Wang Y. et al., 2023). By detecting tumor biomarkers related to BC exosomes (i.e., PMSA, EGFR, CD81, and CEA), FEMC analysis could classify various BC mouse model samples and clinical BC samples within 1 h. Therefore, this method can provide timely and more informed opportunities for clinical BC diagnosis and classification. Although electrochemical methods have many advantages, their disadvantages cannot be ignored, such as surface functionalization challenges, sample matrix effects, and poor reproducibility. Efforts to optimize this strategy are focused on exploring robust methods for surface functionalization and preparing stable electrodes.

Tumor-derived exosomes carry disease-specific protein biomarkers, and their quantitative analysis can significantly enhance diagnostic accuracy. Recent advancements in microfabrication have led to the development of multi-channel electrode architectures with tailored materials and geometries, substantially expanding biosensing capabilities. While single-analyte electrochemical biosensors excel in specificity and operational simplicity for precise biomarker detection, their limited throughput hinder multiplexed analysis. Emerging multiplexed platforms address these limitations by enabling parallel biomarker detection, offering enhanced diagnostic comprehensiveness and compatibility with chip-based portable systems. However, the development of electrochemical biosensors is still constrained by their technical complexity, risks of signal interference, and insufficient clinical validation. In the future, it is necessary to optimize probe design (e.g., anti-interference signal labels), develop stable surface modification techniques, and integrate multi-channel designs (e.g., array chips) or artificial intelligence-assisted analysis to facilitate electrochemical biosensors translation from the laboratory to clinical applications in the field of exosome detection.

## 5 Electrochemical biosensors for the detection of exosomal miRNA

In the past few years, considerable efforts have been made to develop analytical assays for the quantitative detection of exosomal miRNA. Due to the advantages of high sensitivity, simplicity of procedure, and rapid time of detection, biosensors (especially electrochemical l biosensors) has become a promising and useful analysis tool for detection of exosomal miRNA. Although these techniques are influential and powerful, they still have drawbacks and limitations. In the following section, we will summarize the electrochemical biosensors for exosomal miRNA detection in terms of signal amplification. Table 3 summarizes the recent advances in these biosensors for the detection of exosomal miRNA with the focus on the detection method, exosome isolation approach, detection target and detection limit.

**TABLE 3:** Comparison of these electrochemical biosensors for exosomal RNA detection.

Amplification strategy	Exosome isolation approach	Disease	Target protein	LOD	References
Nucleic acid mediated	Ultracentrifugation	Coronary heart disease	miRNA181	7.94 fM	Zhang et al. (2021b)
Nucleic acid mediated	Ultracentrifugation	Breast cancer	miRNA1246/221/375/21	7.2 aM	Zhang et al. (2021c)
Nucleic acid mediated	Selectively captured	MCF-7	miRNA 21	67 aM	Zhang et al. (2018b)
Nucleic acid mediated	Ultracentrifugation	MCF-7	miRNA 21	2.3 fM	Luo et al. (2020)
Nucleic acid mediated	Ultracentrifugation	MCF-7	miR-122	53 aM	Guo et al. (2020)
Nucleic acid mediated	Microfluidic	PANC1 cell	miRNA 550	2 pM	Taller et al. (2015)
Nucleic acid mediated	Ultracentrifugation	Lung cancer	miRNA-155, miRNA-21	33.4 aM, 23.1 aM	Yang et al. (2022)
Enzyme mediated	Ultracentrifugation	Breast cancer	miRNA 21	5.4 fM	Zhang and He (2024)
Enzyme mediated	Ultracentrifugation	Breast cancer	miR-1246	50 aM	Xiao et al. (2024)

The development of electrochemical biosensors for exosomal miRNA detection is an attractive option for fast, cost-effective, and amplification-free applications. Electrochemical technology will probably also play an important role in exosome diagnostics. Consequently, considerable number of electrochemical biosensors have been designed to monitor the exosomal miRNA. Convenient and highly sensitive analysis strategies for exosomal miRNA in electrochemical biosensors is the key to the clinical application of sensors. Therefore, we first summarize existing nucleic acid-based signal amplification strategies.

### 5.1 Nucleic acid mediated signal amplification strategy

**FIGURE 7:**
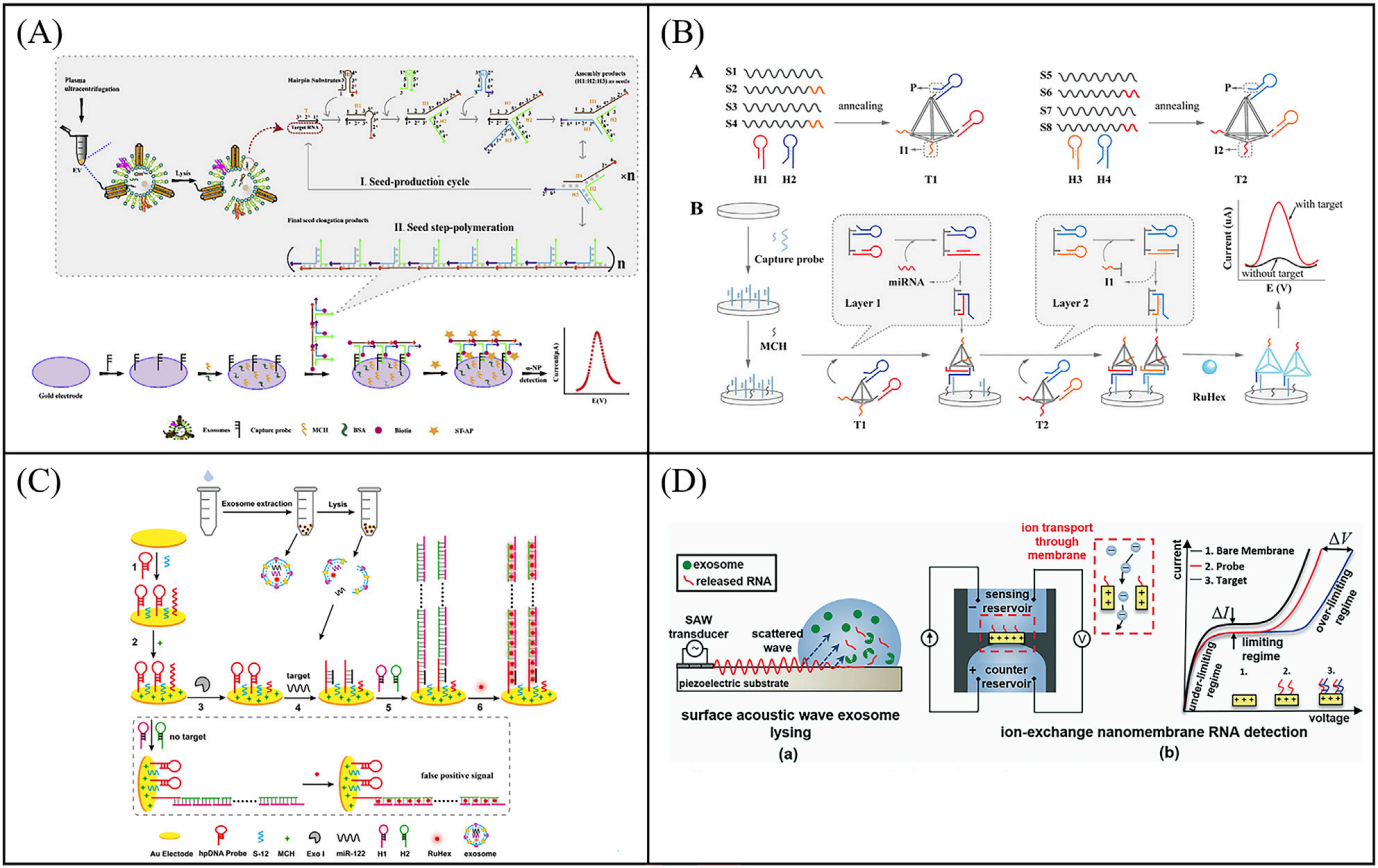
Examples of electrochemical biosensor based on Nucleic acid signal amplification technology for exosomal RNA detection. **(A)** An electrochemical biosensor based on step polymerization catalyzed hairpin assembly (SP-CHA) circuit for the detection of exosomes miR-181. Reprinted with permission from Ref. Zhang et al. (2021b); Copyright (2021) with permission from Elsevier. **(B)** A electrochemical biosensor for exosomal miRNA analysis using multifunctional DNA tetrahedron-assisted catalytic hairpin assembly (MDTs-CHA). Reprinted with permission from Ref. Zhang et al. (2021c); Copyright (2021) with permission from Elsevier. **(C)** A label-free and sensitive electrochemical assay for exosomal miRNA detection based on HCR signal amplification. Reprinted with permission from Ref. Guo et al. (2020); Copyright (2020) with permission from American Chemical Society. **(D)** A microfluidic system consisting of a lysis device by surface acoustic waves (SAW) and a separate detection device for detection of exosomal mRNA. Reprinted with permission from Ref. Taller et al. (2015); Copyright (2015) with permission from Royal Society of Chemistry.

Nucleic acid signal amplification technology is widely used in electrochemical biosensors, which can significantly improve the sensitivity of the sensor. For instance, Zhang et al. designed a new type of electrochemical biosensor based on step polymerization catalyzed hairpin assembly (SP-CHA) circuit for the detection of exosomes miR-181 (Figure 7A) (Zhang R. Y. et al., 2021). The exosomal miR-181 acted as a trigger to initiate the SP-CHA process, resulting in a large number of T-shaped concatemers of different lengths on the electrode surface. The linear range of such biosensor was from 10 fM to 100 nM, and the detection limit was 7.94 fM, which can greatly improve the signal-to-noise ratio. In order to improve the sensitivity of the exosomal microRNA detection, Zhang et al. proposed a robust electrochemical biosensor for exosomal miRNA analysis using multifunctional DNA tetrahedron-assisted catalytic hairpin assembly (MDTs-CHA) (Figure 7B) (Zhang Y. et al., 2021). MDTs-CHA contained two multifunctional tetrahedrons, which used local reactions and cascade amplification for rapid and ultra-sensitive exosomal miRNA analysis. Using MDTs-CHA, the electrochemical platform was able to determine as low as 7.2 aM exosomal miRNA within 30 min with good specificity. Furthermore, the electrochemical biosensor was susceptible to external interference and generate false positive signals during the detection process. For the purpose of improving the accuracy of electrochemical biosensor, Zhang et al. developed a ratiometric electrochemical biosensor based on bipedal DNA walkers for the attomolar detection of exosomal miR-21^78^. Through the signal cascade amplification of DNA walker, the biosensor shows ultra-high sensitivity with a detection limit (LOD) as low as 67 a.m.

In spite of the majority improvement in detection of exosomal miRNA, such strategy still suffered from its practicality due to the additional use of DNA probes and the relatively complex method of strand displacement. To solve this problem, Luo et al. created a Y-shaped structure based on the binding of the MB-signal strand to Fc-signal strand on the electrode surface (Luo et al., 2020). Locked nucleic acid (LNA)-assisted toehold mediated strand displacement reaction (LSDR) happened in the precence of target exosomal miRNA, and Y-shaped-like structure converted into a hairpin structure, turning on the MB signal and turning off the Fc signal. Through ratiometric readout, the electrochemical detection for exosomal miR-21 was realized with an LOD of 2.3 fM. Hybridization chain reaction (HCR) is another signal amplification technique based on DNA strand displacement reaction. In the HCR system, the target molecule triggers two kinds of DNA stem-loops to open alternately, and self-assembles to obtain a linear double-stranded DNA nanostructure containing a large number of repeating units, which has the advantages of constant temperature, enzyme-free, and high amplification efficiency. Guo et al. developed a label-free and sensitive electrochemical assay for exosomal miRNA detection based on HCR signal amplification (Figure 7C) (Guo et al., 2020). The detection limit of the electrochemical assay was greatly improved to 53 aM for miR-122 detection. Overall, electrochemical biosensors based on nucleic acid signal amplification greatly improve the sensitivity of exosomal RNA detection and provide more accurate and precise results for cancer diagnosis.

In order to develop more portable sensing devices to meet the clinical detection needs of cancer, combining the advantages of microfluidic technology and electrochemical technology, Taller et al. developed a microfluidic system consisting of a lysis device and a separate detection device for detection of exosomal miRNAs (Figure 7D) (Taller et al., 2015). MiRNAs were released from exosomes cleaved by surface acoustic waves (SAW) and captured by miRNA probes through ion-exchange nanofilms. The entire procedure took only ∼1.5 h, which included ∼30 min for lysing and ∼1 h for detection. At the same time, the sensor shown a limit of detection of 2 pM. SAW lysis has more benefits than typical chemical lysis methods, opening up additional options for exosomal miRNA detection on a microfluidic device based on electrochemical technology. In order to further improve the throughput of miRNA detection, multi-target detection platforms are gradually emerging. Yang et al. proposed a disposable paper-based electrochemical strategy based on nucleic acid functionalized Zr MOF, which was label free and enzyme free, and could simultaneously detect miRNAs in multiple T-EXOs (Yang et al., 2022). This biosensor eliminated multiple time-consuming steps associated with existing miRNA quantification methods, such as reverse transcription and thermal cycler amplification. And this method achieved molecular level sensitivity detection of exosomal miRNA-155 (33.4 aM) and exosomal miRNA-21 (23.1 aM). In addition, the disposable paper biosensing strategy used in this sensor provides more possibilities for developing flexible, convenient, easy-to-use, and inexpensive bioelectrodes.

### 5.2 Enzyme mediated signal amplification strategy

**FIGURE 8:**
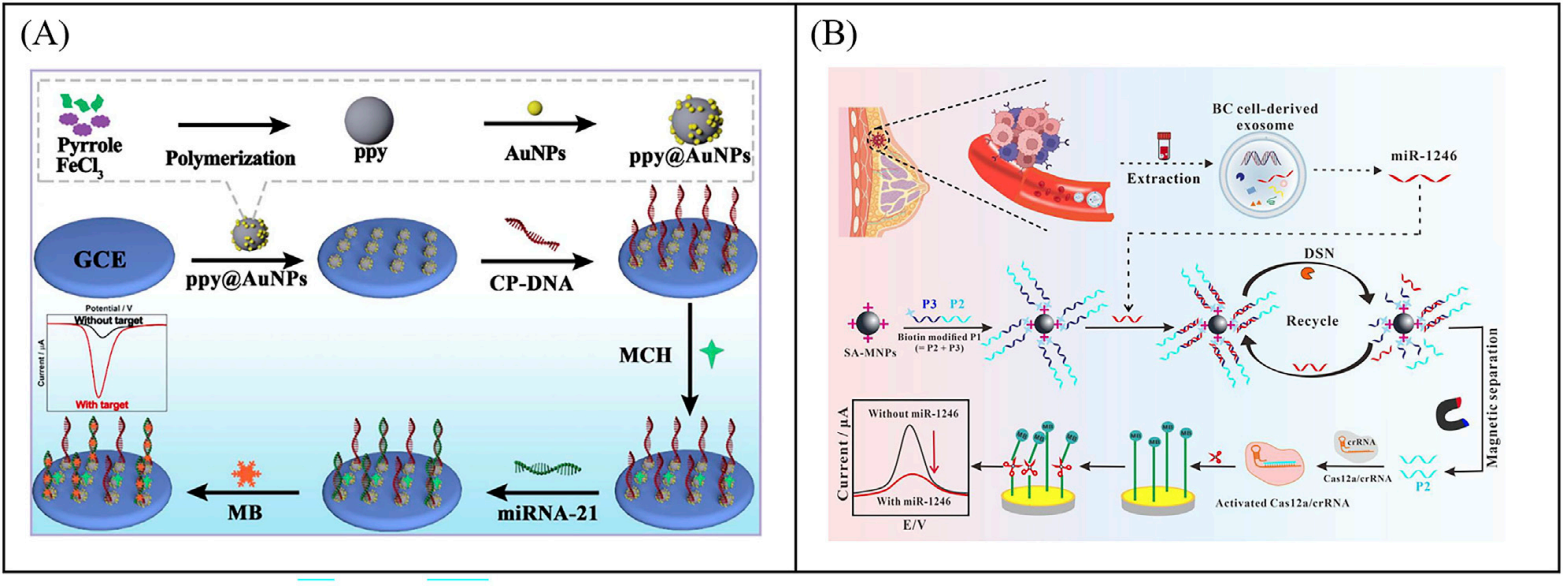
Examples of electrochemical biosensor based on enzyme mediated signal amplification technology for exosomal RNA detection. **(A)** An electrochemical biosensor to detect miRNA-21 using a signal amplification strategy mediated by polypyrrole@gold nanocomposites, resulting in the development of an ultra-sensitive electrochemical biosensor. Reprinted with permission from Ref. Zhang and He (2024); Copyright (2024) with permission from Royal Society of Chemistry. **(B)** Combining CRISPR technology with electrochemical biosensor based on CRISPR/Cas12a-DSN-MNPs for detection of exosomal miR-1246. Reprinted with permission from Ref. Xiao et al. (2024); Copyright (2024) with permission from Elsevier.

In electrochemical sensors, nanomaterials as electrocatalysts show excellent performance for the detection of biological samples. Zhang et al. developed an ultra-sensitive electrochemical biosensor to detect miRNA-21 using a signal amplification strategy mediated by nanoenzyme (Zhang and He, 2024). The polypyrrole@gold nanocomposites (ppy@AuNPs) were used as electrochemical catalysts to enhance signal amplification (Figure 8A). The electrochemical platform showed analytical performance with a wide linear range from 10 fM to 100 nM and low detection limits as low as 5.4 fM.

CRISPR (clustered regularly interspaced short palindromic repeats) technology is a precise genome editing technique. In the CRISPR system, guide RNA guides nucleases to bind to specific nucleic acid sequences and cleave them, which has the advantages of high sensitivity, strong specificity, mild reaction temperature, and fast reaction speed (Yan and Li, 2019). The CRISPR Cas system includes Cas9, Cas12a, Cas13a, and so on. Among them, CRISPR/Cas12a not only has cis cutting activity for recognizing and cleaving target DNA, but also has unique trans cutting activity, which can indiscriminately cleave non target single stranded DNA (ssDNA) (Chen et al., 2023). In a typical CRISPR/Cas12a system, programmable crRNA guides Cas12a protein to recognize target DNA and assemble into a triple complex. Then the triple complex activates Cas12a′s efficient trans cleavage activity to cleave surrounding ssDNA signaling reporter genes, providing a new pathway for next-generation molecular diagnostic techniques (Chen et al., 2023). Xiao et al. combined CRISPR technology with electrochemical sensors to develop an electrochemical biosensor based on CRISPR/Cas12a, double stranded specific nucleases (DSN), and magnetic nanoparticles (MNPs) for detecting miR-1246 in BC derived exosomes (Figure 8B) (Xiao et al., 2024). The target specificity of DSN and Cas12a systems triggered enzyme digestion activity. The strong separation ability of MNPs ensured that the developed electrochemical biosensor had high specificity in distinguishing single base mismatches, which could cope with the challenges of low abundance, high homology, and complex background interference in miR-1246 detection. The sensor had been successfully applied for the detection of bloodborne exosomal miR-1246, achieving differentiation between BC patients and healthy individuals. Although enzyme-mediated signal amplification strategies have demonstrated excellent performance in the detection of exosomal miRNA, enzymes with high catalytic performance and high stability still need to be further developed.

Electrochemical biosensors offer distinct advantages for exosomal miRNA analysis, including rapid detection kinetics (<30 min) and femtomolar-level sensitivity. Nucleic acid-based amplification strategies have enabled ultraspecific exosomal miRNA detection, with enzyme-free configurations and portable designs enhancing clinical feasibility. Nevertheless, challenges in multiplex assay reliability, false-positive mitigation, and technical reproducibility need to be addressed. In the future, it is essential to develop more stable probe immobilization techniques, anti-interference signal amplification strategies, and streamline the process for simultaneous multi-target detection to enhance the practicality of electrochemical sensors in exosome detection. While enzyme-mediated amplification systems demonstrate superior sensitivity and clinical utility, they require optimization in terms of operational stability and device independence. The development of simplified reaction schemes, durable nanozyme materials, and field-deployable devices could facilitate their transformation into clinical point-of-care diagnostics.

## 6 Comparison of electrochemical biosensors and conventional methods in detecting exosomes

Traditional methods for exosome quantification often suffer from limitations such as poor specificity, low sensitivity, prolonged detection times, high costs, large sample requirements, and lack of portability. In contrast, electrochemical biosensors, when integrated with the high specificity of aptamers/antibodies, enable highly specific detection of tumor exosomes. Furthermore, by incorporating advanced signal amplification strategies, these sensors can achieve single-exosome analysis. The integration of microfluidic technology and array chips has significantly reduced detection times and minimized sample volumes, further enhancing their practicality.

Table 4 summarizes the comparison of electrochemical biosensors and conventional methods in detecting exosomes. Traditional methods for detecting exosomal proteins often demand large sample volumes, involve time-consuming procedures (ranging from 1 to 10 hours), and are constrained by limited sensitivity, resolution, and the high cost of equipment. Electrochemical biosensors provide significant advantages, such as minimal sample requirements, fast detection, user-friendly operation, and the capability to achieve highly sensitive detection of low-abundance proteins. Nevertheless, their throughput remains a constraint. To overcome this, future advancements could emphasize the integration of multi-channel designs (e.g., array chips) or the application of artificial intelligence-assisted analysis to facilitate high-throughput, multiplexed biomarker detection, thereby enhancing the precision of cancer diagnosis.

**TABLE 4:** Comparison of electrochemical biosensors and conventional methods in detecting exosomes.

Item	Indicator	Traditional detection methods	Electrochemical biosensors
Quantification of Exosome Numbers	Sensitivity	10^5^–10^7^ particles/μL	0.5–20 particles/μL
	Specificity	Medium - high (dependent on labels)	High
	Detection Time	30–60 min	10–20 min (microfluidic integration)
	Cost	High (equipment/reagents)	Low (paper - based)
	Sample Requirement	10–500 μL	1–10 μL
	Portability	Low (large equipment)	High (portable)
Detection of Exosomal Proteins	Detection Time	Long (1–10 h)	Short (within 1 h)
	Operation Complexity	Complex	simple
	Detection Limit	Usually high detection limit	lower detection limit
	Cost	High (equipment)	Low
	Sample Requirement	Large (μg)	Low (10 μL plasma)
	Portability	Low (large equipment)	High (portable)
Detection of Exosomal miRNA	Sensitivity	pM-fM level	aM level
	Detection Time	Hours-days	30 minutes-1.5 h
	Stability	RT-PCR is vulnerable to amplification contamination; miRNA microarray has weak ability to distinguish homologous sequences.	The anti-interference performance needs to be improved
	Operation Complexity	Northern blot has standardized procedures but low efficiency; RT-PCR requires professional equipment and technical personnel	Electrochemical methods still need to simplify probe design and reaction steps
	Cost	High (equipment/reagents)	Low

For exosomal miRNA detection, traditional methods are frequently limited by their high costs, intricate procedures, slow detection speeds, susceptibility to interference, and elevated risk of false positives. These constraints hinder their widespread use in exosome-related research. In contrast, electrochemical biosensors, with their inherent strengths of high sensitivity, simplicity, rapid detection, and cost-effectiveness, have emerged as a highly promising alternative for exosomal miRNA analysis.

In summary, electrochemical biosensors exhibit substantial potential in the detection of tumor exosomes, providing a versatile and efficient platform that overcomes many limitations of traditional methods. We anticipate that these advancements will significantly enhance exosome research and its clinical applications.

## 7 Conclusions and perspectives

Exosomes play an important role in the genesis and development of tumors and are considered as novel, potential biomarkers. Exosome-based liquid biopsy has shown great potential for cancer diagnostics and therapeutics. Electrochemical biosensors are easy to miniaturize and integrate, which is conducive to the development of portable devices. Electrochemical biosensors provide a kind of sensing way with high sensitivity, fast response and simple operation for the analysis of biological samples. The review provides an in-depth description of the construction of various electrochemical biosensors for the detection of exosomes and exosome derived biomarkers. It is worth noting that microfluidic chip technology has shown great advantages in exosome enrichment from complex samples and analysis due to high throughput, automation, miniaturization and easy integration of downstream analysis. However, exosome-based liquid biopsy still faces a number of critical challenges for cancer diagnostics. Therefore, the following perspectives are proposed for the analysis of exosomes.

### 7.1 Development of electrochemical microfluidic chips

Electrochemical technology has the advantages of being relatively easy to use, highly sensitive and miniaturized. Microfluidics can handle or manipulate tiny fluids of picoliter to microliter by designing microchanels or microchambers, offering compelling advantages in separation and detection of exosomes. Overall, microfluidics-assisted electrochemical biosensors can combine the advantages of microfluidics and bisensing for efficient, highly sensitive, high selective detection of exosomes. At present, several microfluidic platforms based electrochemical sensing have been used for the detection of exosomes. Even so, we find that electrochemical-based microfluidic chips are still relatively rare for cancer diagnosis. Designing fine electrode structures on microfluidic chips will effectively improve the performance of sensor. The design of detachable electrochemical microfluidic device can greatly improve the reusability of chips. Furthermore, optimizing the cleavage scheme of exosomes can facilitate the analysis of exosome RNA *in situ*. Biosensors combining the advantages of electrochemistry and microfluidic chips will open up an additional field of study for exosome detection.

### 7.2 Single exosome analysis

Exosomes secreted by different cells differ in their structure and composition, leading to the existence of many subtypes of exosomes. In particular, the evaluation of individual exosomal proteins and miRNA has important research significance for the diagnosis and prognosis of tumors. However, the detection of a high-throughput single exosome still faces significant challenges due to lack of adequately accurate and sensitive assay platforms. Therefore, the development of a high-throughput single exosome detection platform combined with coding strategies and machine learning-assisted bioinformatics analysis will significantly enhance the importance of exosomes in clinical analysis.

### 7.3 Novel marker detection for exosomes

Exosomes transport various molecular contents of the cell from which they originate, including lipids, proteins and nucleic acids. It has been shown that tumor exosomes differ significantly from normal exosomes in terms of the lipid structure and hardness. Meanwhile, tumor-derived exosomes are richer in dsDNA. Currently, the protein analysis of exosomes is focused on tumor markers, such as CD24, CD125, HER2, EpCAM, PSMA, CEA and so on. However, the existence of other tumor marker proteins is unknown. Therefore, the development of sensitive sensing platforms for the screening and analysis of novel exosome tumor markers is crucial to a comprehensive study and to the therapeutic use of exosome.

### 7.4 Integration of artificial intelligence (AI) in exosome analysis

The integration of AI and machine learning (ML) into exosome analysis holds immense potential for revolutionizing cancer diagnostics and personalized medicine. AI algorithms can process vast amounts of complex data generated from exosome profiling, including proteomic, genomic, and lipidomic data, enabling the identification of novel biomarkers and patterns that may not be discernible through traditional analytical methods. AI can also facilitate the automated classification and sorting of exosomes based on their molecular signatures, improving the accuracy and efficiency of exosome isolation. Furthermore, AI-driven predictive models can correlate exosome profiles with disease states, progression, and treatment responses, paving the way for non-invasive diagnostic tools and personalized therapeutic strategies. The combination of AI with portable biosensor devices could enable real-time monitoring of exosome levels and profiles, providing continuous insights into disease progression and treatment efficacy.

### 7.5 Advancements in portable biosensor devices

The development of portable biosensor devices is a critical step toward the widespread clinical adoption of exosome-based diagnostics. Advances in portable biosensors, particularly those integrated with microfluidic and electrochemical technologies, offer the potential for point-of-care (POC) testing in resource-limited settings. Portable biosensors can enable rapid, on-site detection of exosomes, reducing the need for complex laboratory equipment and lengthy processing times. Wearable or implantable biosensors could provide continuous, real-time monitoring of exosome levels in bodily fluids, offering valuable insights into chronic disease management and treatment responses. The miniaturization of biosensors, combined with wireless connectivity and cloud-based data analysis, could further enhance their utility in remote and decentralized healthcare settings.

To overcome the existing challenges and fully harness the potential of exosome-based diagnostics, future research should prioritize the development of advanced electrochemical microfluidic chips, high-throughput single exosome analysis platforms, and sensitive sensing technologies for novel marker detection. The integration of AI and machine learning into exosome analysis will be pivotal for unlocking new diagnostic and therapeutic insights. Additionally, the advancement of portable biosensor devices will enable real-time, point-of-care testing, making exosome-based diagnostics accessible in diverse clinical settings. Collaborative efforts across disciplines will be essential to drive innovation and translate these technologies into practical clinical applications, ultimately improving cancer diagnostics and personalized medicine.
